# Tightrope vs. hook plate fixation for acute acromioclavicular joint dislocation: a systematic review and meta-analysis

**DOI:** 10.1016/j.xrrt.2025.04.002

**Published:** 2025-04-30

**Authors:** Brandon Lim, Ariel Chai, Samher Jassim, Mohamed Shaalan

**Affiliations:** a Department of General Surgery, Sengkang General Hospital, Singapore, Singapore; b Department of Trauma and Orthopaedic Surgery, Tallaght University Hospital, Dublin, Ireland

**Keywords:** TightRope, Clavicular hook plate, Acromioclavicular joint, Clavicle, Dislocation, Surgical treatment

## Abstract

**Background:**

Treatment for acromioclavicular joint (ACJ) dislocations aims to restore joint congruity and mechanical stability. However, the best operative technique remains a controversial issue. This systematic review and meta-analysis thus aim to compare the clavicular hook plate (HP) vs. the TightRope (TR) in the management of ACJ dislocation.

**Methods:**

A systematic search was conducted using Embase, Scopus, PubMed, and Web of Science databases to retrieve all relevant studies. Outcomes were operative time (minutes), intraoperative blood loss (mL), clinical outcome measures, postoperative coracoclavicular distance (CCD), and complications. The methodological quality of studies was assessed using the Methodological Index for Nonrandomized Studies tool for nonrandomized studies, and the Cochrane Risk of Bias 2 tool for randomized control trials.

**Results:**

The literature search yielded 221 studies, of which 12 studies enrolling a total of 683 patients were included in this review, with 371 in the HP group and 312 in the TR group. Meta-analysis of comparative studies between HP and TR fixation showed that HPs had better Constant-Murley scores (mean difference (MD), −3.56; 95% confidence interval (CI), −5.37 to −1.75; *P* = .0001), and less intraoperative blood losses (MD, 41.27; 95% CI, 30.67-51.87; *P* < .00001). Conversely, TR fixation had better visual analog scale scores (MD, 0.55; 95% CI, 0.34-0.76; *P* < .0001), and shorter postoperative CCD (MD, 0.45; 95% CI, 0.19-0.71; *P* = .0008). There was no significant difference in operative time (MD, 1.75; 95% CI, −16.55-20.05; *P* = .85), University of California, Los Angeles shoulder scores (MD, 0.34; 95% CI, −0.81 to 1.48; *P* = .56), American Shoulder and Elbow Surgeons scores (MD, 0.39; 95% CI, −0.90 to 1.68; *P* = .55), and complications (OR, 2.57; 95% CI, 1.00-6.62; *P* = .05).

**Conclusion:**

TR fixation in ACJ dislocations had similar operative times, complication rates, University of California, Los Angeles scores, and American Shoulder and Elbow Surgeons scores to HP fixation. The HP group had less intraoperative blood loss and better Constant-Murley scores. Conversely, TR fixation had better visual analog scale scores and smaller postoperative CCD. Future randomized control trials on this subject would aid in increasing the validity of our findings.

Acromioclavicular joint (ACJ) dislocations account for 12% of shoulder injuries,^25^^,^^32^ occur 5 times more in men than women, and have peak incidence in 20- to 30-year-olds.^1^ ACJ dislocations are commonly seen in athletic injuries or falls on an outstretched hand, athletic injuries accounting for nearly half of all ACJ dislocations.^32^ These injuries occur when a direct force exerted on the acromion with an adducted shoulder moves the acromion while the clavicle remains stabilized by sternoclavicular ligaments.^1^^,^^25^

The goal of treatment for ACJ dislocations is to restore the coracoclavicular distance (CCD) and allow for ruptured ligaments to heal.^25^ The choice of treatment for ACJ dislocations depends on the Rockwood classification with grade I, II, and uncomplicated grade III injuries being managed nonoperatively, and higher-grade dislocations requiring surgery.^1^^,^^4^ Several techniques have been used to treat ACJ dislocations by restoring joint congruity and mechanical stability but the best operative technique remains a controversial issue.^1^^,^^2^^,^^6^^,^^14^^,^^17^^,^^25^ A biomechanical study by Lädermann et al. comparing the hook plate (HP), TightRope (TR) (Arthrex, Naples, FL, USA), and coracoclavicular cerclages reported that the TR provides the most stability while the HP provided no biomechanical advantages compared with the other 2 techniques.^14^ Another biomechanical study by Nüchtern et al. comparing the HP, TR, and bone anchor systems reported that the HP provided axial stiffness closest to normal physiology while the TR provided a higher vertical load capacity.^17^

Meta-analyses comparing HPs to suture buttons, both TRs and Endobuttons (Smith & Nephew, Andover, MA, USA) have been published in the literature.^9^^,^^29^ However, since the TR can be considered an upgrade to the Endobutton system and some studies suggest the TR to be advantageous to the Endobutton,^11^^,^^28^ we have chosen to only include studies that compare the TR to the HP in our review. This systematic review and meta-analysis thus aims to compare the clavicular HP vs. TR in the management of ACJ dislocation.

## Methods

This systematic review and meta-analysis was conducted using the Preferred Reporting Items for Systematic Reviews and Meta-Analyses guidelines.^18^

### Eligibility criteria

All observational studies written in English and comparing clavicular HPs vs. TR in ACJ dislocation were included. Nonoriginal studies, noncomparative studies, case reports, non-English articles, and non–full-text articles, were excluded. No restrictions on follow-up time were applied.

### Outcomes

Outcomes were operative time (minutes), intraoperative blood loss (mL), clinical outcome measures, postoperative CCD, and complications.

### Literature search strategy

A systematic search was performed in Embase, Scopus, PubMed, and Web of Science from their respective inceptions to October 1, 2024. The following keywords were used: TR AND (HP OR bone plate) AND acromioclavicular. Search results were imported into the Covidence online software tool and duplicates were automatically removed. This search was carried out by 2 independent authors.

### Study selection

Two authors independently assessed titles and abstracts. Full texts of relevant articles were retrieved, and those that met the inclusion criteria were selected. Discrepancies were resolved by discussion with a third reviewer.

### Data extraction

Data extracted onto Excel Sheets included authors, year of study, study design, study size, Rockwood classification of ACJ dislocation, patient characteristics, and the outcomes of interest.

### Data analysis

Data were analyzed using RevMan Web (Cochrane Collaboration, London, UK). Mean differences (MDs) and 95% confidence intervals (CIs) were pooled for continuous outcomes. Odds ratios (ORs) with 95% CIs were employed using the Mantel-Haenszel method for dichotomous outcomes. Acceptable heterogeneity between included studies was defined as I^2^ ≤ 50% and *P* > .1. Under this circumstance, a fixed-effects model was applied; otherwise, a random-effects model was applied. Statistical significance was defined as *P* < .05.

### Quality assessment

Two reviewers independently assessed the methodologic quality of all included studies using the Methodological Index for Nonrandomized Studies (MINORS) tool for nonrandomized studies,^24^ and the Cochrane Risk of Bias 2 tool for randomized studies.^10^ Disagreements were resolved by involving a third reviewer. The MINOR tool for comparative studies assigns 0-2 points to each of its 12 criteria, resulting in a maximum score of 24, with 0-6 indicating very low-quality evidence; 7-10 indicating low-quality evidence, 10-16 indicating fair-quality evidence, and >16 indicating good quality evidence.^24^ For randomized control trials (RCTs), the Cochrane Risk of Bias 2 tool gave a rating of low, high, or unclear risk for selection bias, performance bias, detection bias, attrition bias, reporting bias, and other bias based on in-text evidence.^10^

## Results

**Figure 1:**
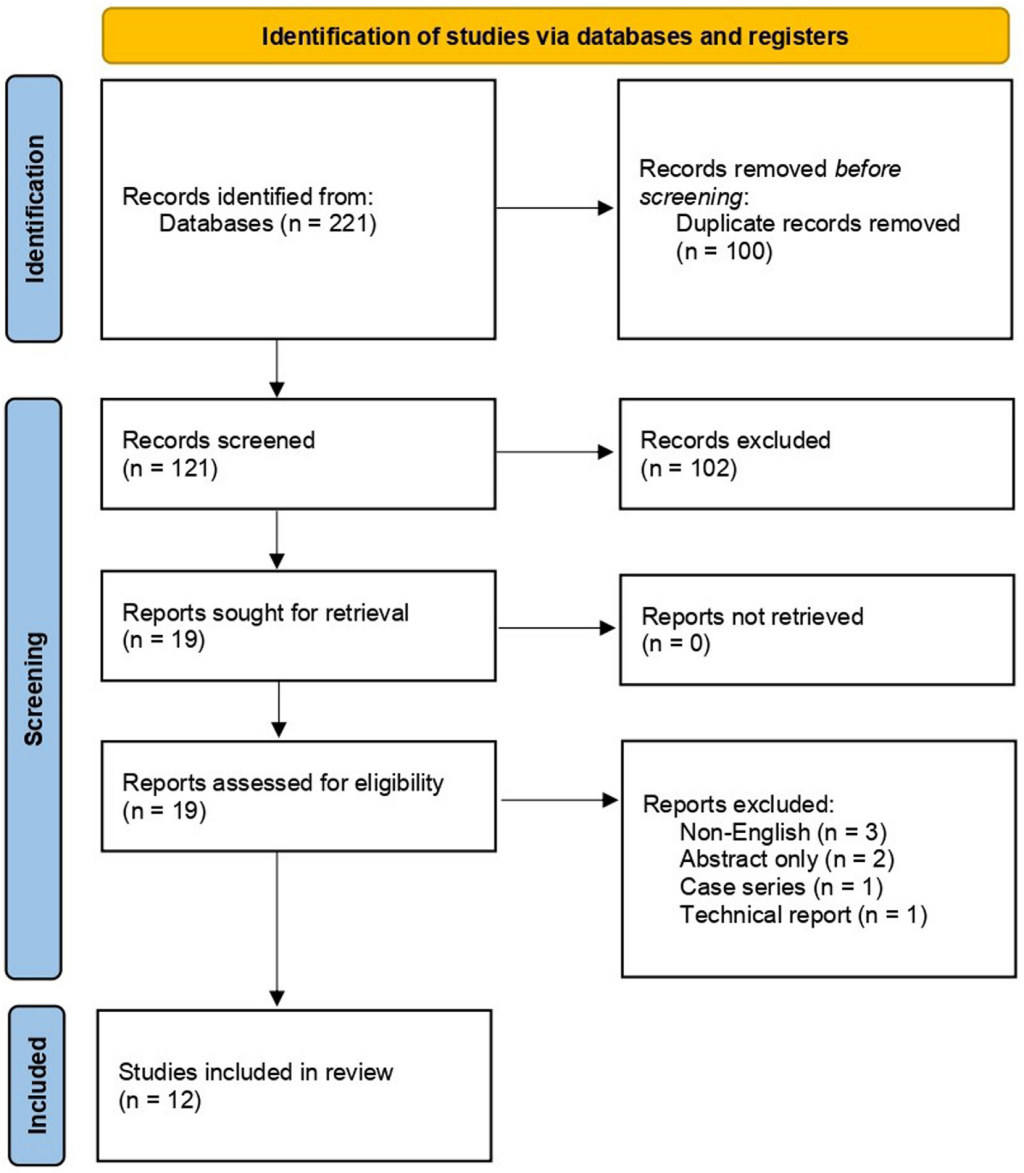
Flowchart of study screening and selection using the Preferred Reporting Items for Systematic Reviews and Meta-Analyses guidelines.

Our search strategy retrieved 221 studies. A thorough screening of retrieved articles yielded 12 studies that met the eligibility criteria (Fig. 1). These 12 studies enrolled a total of 683 patients with 371 in the HP (HP) group and 312 in the TR (TR) group.^3^^,^^5^^,^^7^^,^^8^^,^^13^^,^^16^^,^^20^^,^^22^^,^^23^^,^^26^^,^^27^^,^^31^ Characteristics of the included studies are summarized in Table I.

**Table I:** Summary of included studies.

Author (yr)	Country	Study design	Injury classification, n	Treatment groups	No. of patients, n	Follow-up, mo	Age (mean ± SD), yr	Gender (m/f)	MINORS score (n/24)
Bin Abd Razak et al (2017)^3^	Singapore	Prospective	Rockwood III-V	HP	10	23	49.2 ± 16.9	9/1	21
				TR	16	23	41.4 ± 12.3	15/1	
Cai et al (2017)^5^	China	Randomized control trial	Rockwood III	HP	39	12	41.79 ± 10.21	26/13	NA
				TR	30	12	42.80 ± 11.88	19/11	
Pongsamakthai and Tharakulphan (2018)^20^	Thailand	Randomized control trial	Rockwood III-V	HP	22	3	39.6 ± 9.6	17/5	NA
				TR	22	3	37.1 ± 11.5	16/6	
Nie and Lan (2021)^16^	China	Prospective	Rockwood III-V	HP	84	32.9 + 6.4	36.0 + 8.3	33/51	20
				TR	28	33.1 + 6.0	35.9 + 7.9	11/17	
Shen et al (2021)^22^	China	Retrospective	Rockwood III/V	HP	19	30	40.2 ± 8.7	10/9	16
				TR	16	27	44.9 ± 11	11/5	
Dundar and Ipek (2022)^7^	Turkey	Retrospective	Rockwood III	HP	23	20.1 ± 2.2	39.2 ± 8.7	20/3	16
				TR	21	19.9 ± 3.1	43.9 ± 11	19/2	
Gultac et al (2022)^8^	Turkey	Retrospective	Rockwood III/V	HP	14	12	41.8	13/1	14
				TR	21	12	39.2	19/2	
Thuy et al (2022)^26^	Vietnam	Retrospective	Rockwood IIIb-V	HP	7	-	44.29 ± 9.59	6/1	13
				TR	32	-	41.53 ± 9.72	20/23	
Ko et al (2023)^13^	Korea	Retrospective	Rockwood III-V	HP	36	81.6 ± 12	51.5 ± 13.1	31/5	20
				TR	25	86.4 ± 10.8	47.9 ± 13.3	23/2	
Sheu et al (2023)^23^	Taiwan	Retrospective	Rockwood V	HP	22	30.2 ± 4.3	45.7 ± 12.3	15/7	18
				TR	26	30.2 ± 4.3	42 ± 16.2	20/6	
Yu et al (2023)^31^	China	Retrospective	Rockwood IIIB	HP	60	12	56.43 ± 9.64	35/25	19
				TR	52	12	50.15 ± 15.39	32/20	
Wang et al (2024)^27^	China	Retrospective	Rockwood III/IV	HP	35	15.4	45.86 ± 11.01	27/8	21
				TR	23	15.4	40.57 ± 11.42	13/10	

*HP*, hook plate; *TR*, TightRope; *SD*, standard deviation; *MINORS*, methodological index for nonrandomized studies.

### Operative time (minutes)

**Figure 2:**
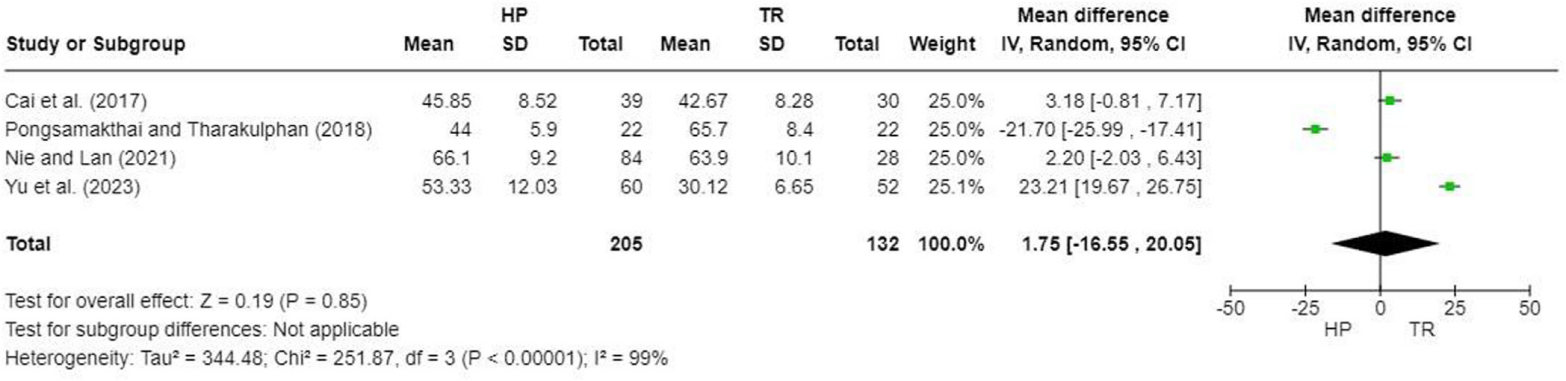
Forest plot of operative time (minutes). *HP*, hook plate; *TR*, TightRope; *SD*, standard deviation.

Operative time was reported in 5 studies,^5^^,^^8^^,^^16^^,^^20^^,^^31^ but one did not report standard deviations.^8^ A meta-analysis performed for 4 studies demonstrated no significant difference in operative time between groups (MD, 1.75; 95% CI, −16.55 to 20.05; *P* = .85) (Fig. 2).

### Intraoperative blood loss (mL)

**Figure 3:**
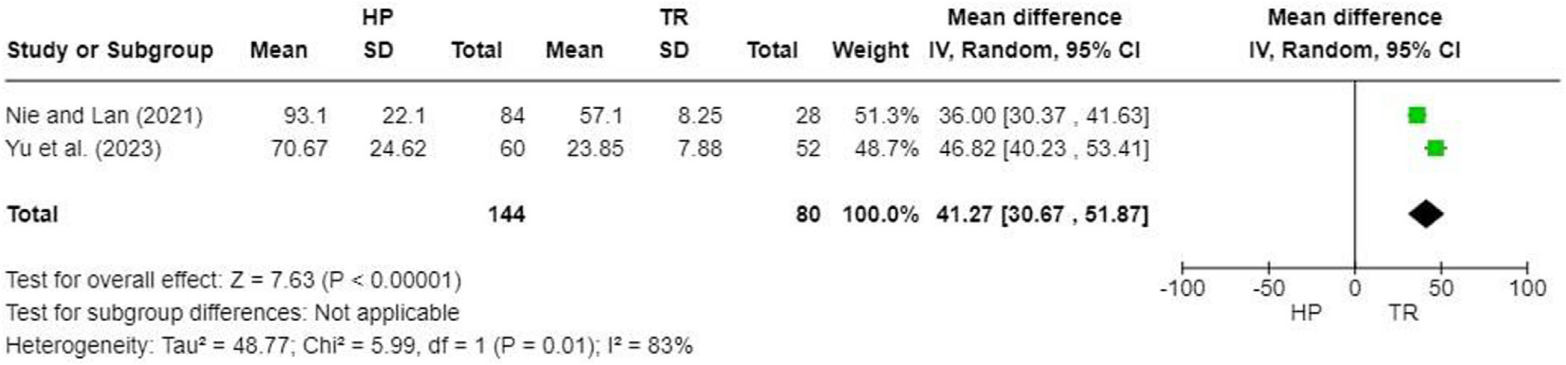
Forest plot of intraoperative blood loss (mL). *HP*, hook plate; *TR*, TightRope; *SD*, standard deviation; *CI*, confidence interval.

Intraoperative blood loss was reported in 2 studies.^16^^,^^31^ There was a significantly lower intraoperative blood loss in TR groups (MD, 41.27; 95% CI, 30.67-51.87; *P* < .00001) (Fig. 3).

### Constant-Murley score

**Figure 4:**
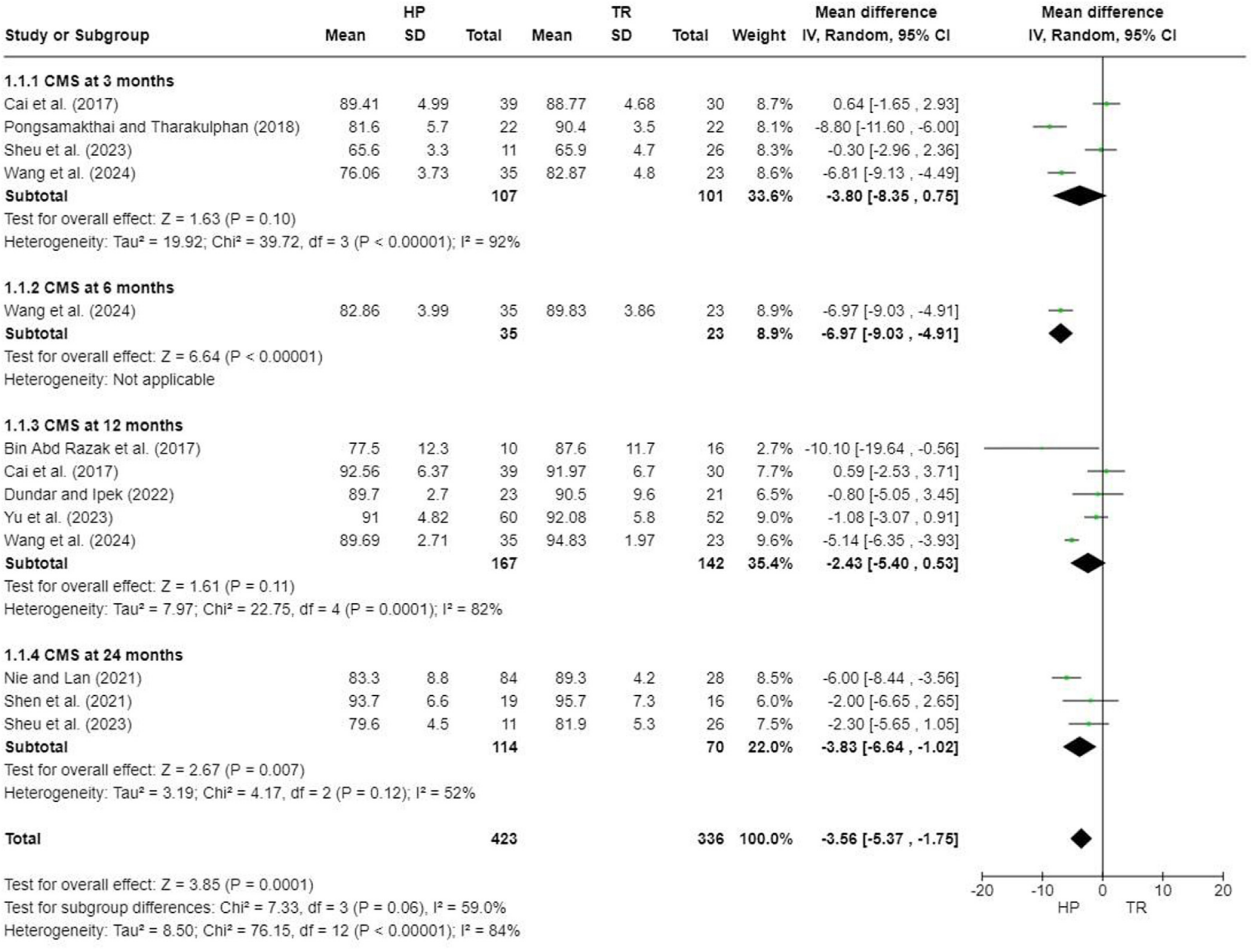
Forest plot of Constant-Murley scores. *HP*, hook plate; *TR*, TightRope; *SD*, standard deviation; *CI*, confidence interval; *CMS*, Constant-Murley score.

Nine studies reported Constant-Murley scores (CMS) at different follow-up times,^3^^,^^5^^,^^7^^,^^8^^,^^20^^,^^23^^,^^26^^,^^27^^,^^31^ but one did not specify the time at which CMS was recorded,^26^ and one did not report standard deviations.^8^ A meta-analysis performed for 7 studies demonstrated a significant difference between the HP and TR groups (MD, −3.56; 95% CI, −5.37 to −1.75; *P* = .0001). There was no significant difference at 3 months after the surgery (MD, −3.80; 95% CI, −8.35 to 0.75; *P* = .10) and 12 months after the surgery (MD, −2.43; 95% CI, −5.40 to 0.53; *P* = .11), but a significant difference was observed at 24 months after the surgery (MD, −3.83; 95% CI, −6.64 to −1.02; *P* = .007) (Fig. 4).

### Visual analog scale

**Figure 5:**
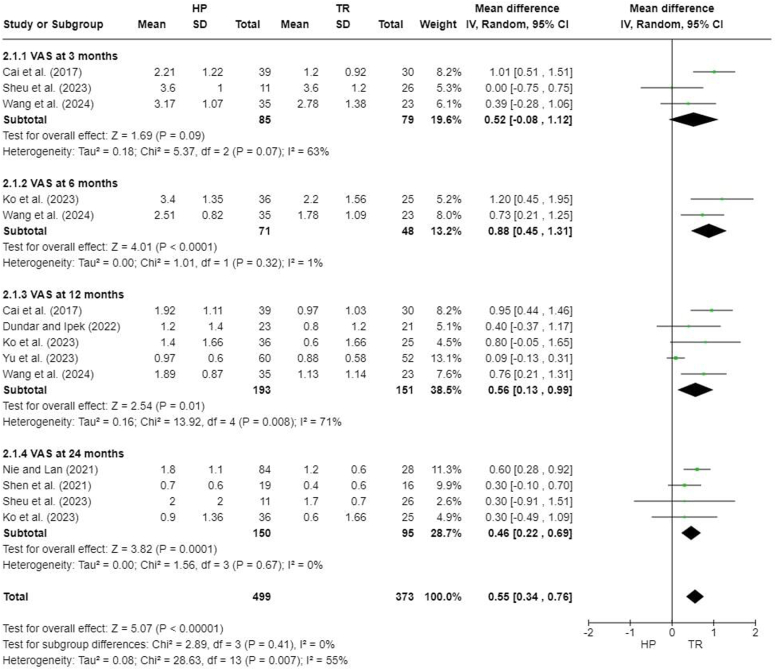
Forest plot of visual analog scale scores. *HP*, hook plate; *TR*, TightRope; *SD*, standard deviation; *CI*, confidence interval; *VAS*, visual analog scale.

Six studies reported visual analog scale (VAS) scores at different follow-up times.^5^^,^^7^^,^^13^^,^^23^^,^^27^^,^^31^ A significant difference was found between the HP and TR groups (MD, 0.55; 95% CI, 0.34-0.76; *P* < .0001). There was no significant difference at 3 months after the surgery (MD, 0.52; 95% CI, −0.08 to 1.12; *P* = .09), but a significant difference was observed at 6 months (MD, 0.88; 95% CI, 0.45-1.31; *P* < .00001), 12 months (MD, 0.56; 95% CI, 0.13-0.99; *P* = .01), and 24 months (MD, 0.46; 95% CI, 0.22-0.69; *P* = .0001) (Fig. 5).

### University of California, Los Angeles shoulder score

**Figure 6:**
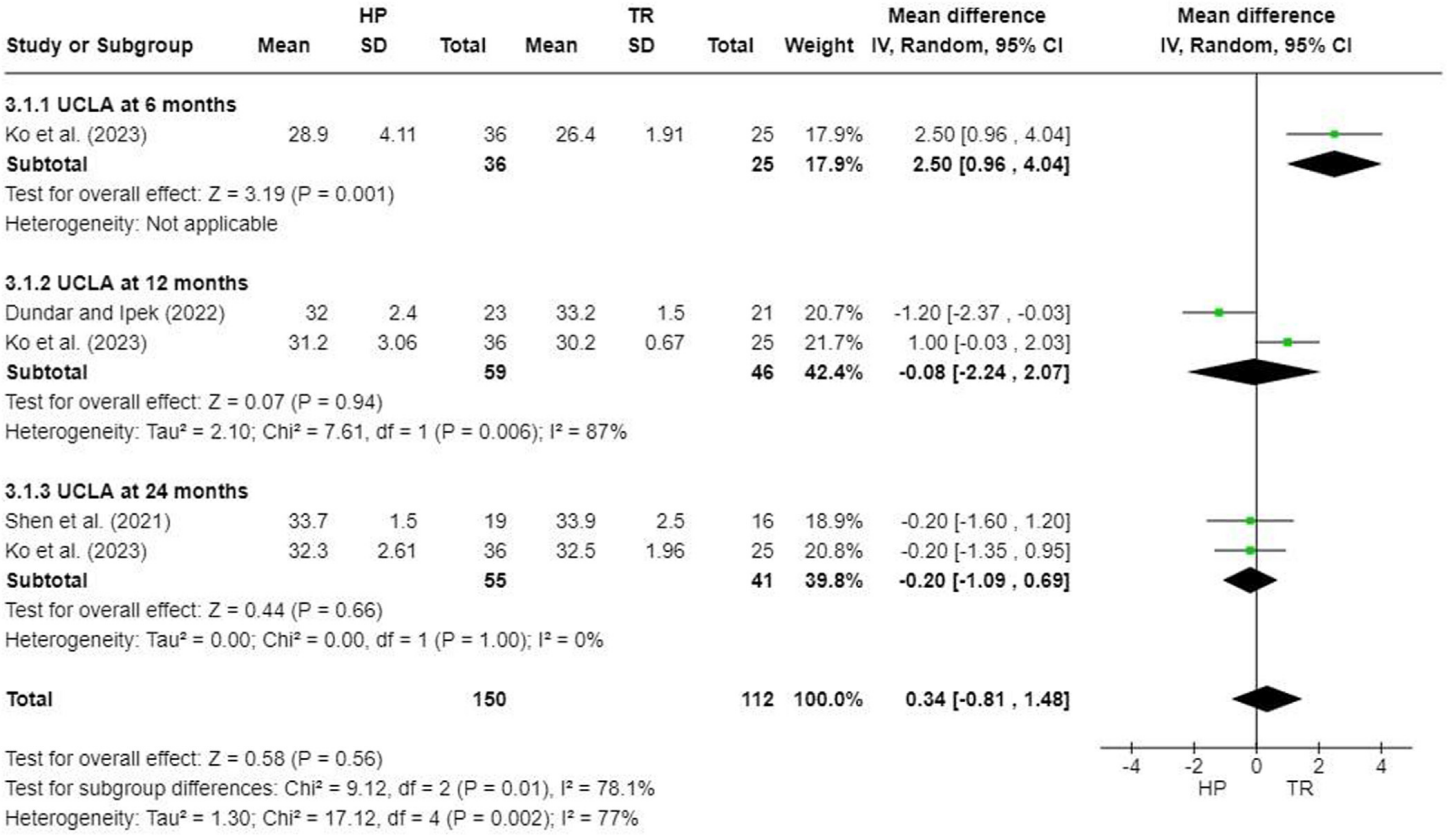
Forest plot of University of California, Los Angeles scores. *HP*, hook plate; *TR*, TightRope; *SD*, standard deviation; *CI*, confidence interval; *UCLA*, University of California Los Angeles.

Three studies reported University of California, Los Angeles (UCLA) scores at different follow-up times.^7^^,^^13^^,^^22^ No significant difference was found between the HP and TR groups (MD, 0.34; 95% CI, −0.81 to 1.48; *P* = .56). There was no significant difference at 12 months (MD, −0.08; 95% CI, −2.24 to 2.07; *P* = .94) or 24 months after surgery (MD, −0.20; 95% CI, −1.09 to 0.69; *P* = .66) (Fig. 6).

### American shoulder and Elbow Surgeons score

**Figure 7:**
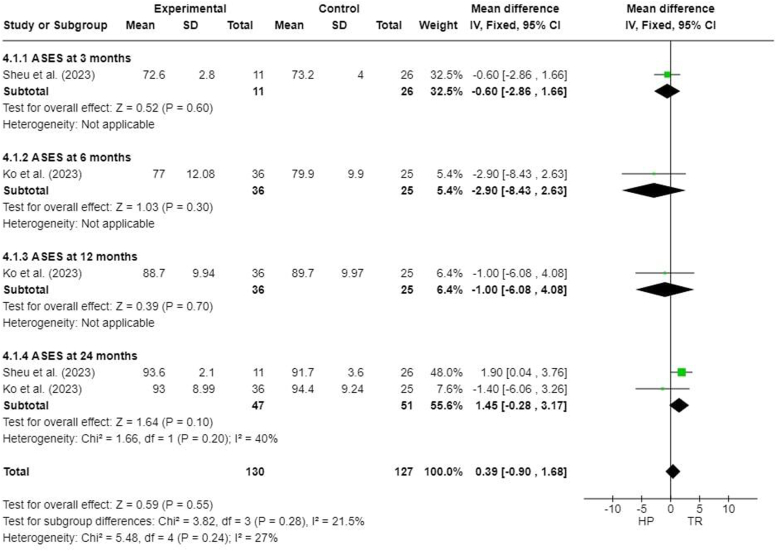
Forest plot of American Shoulder and Elbow Surgeons scores. *HP*, hook plate; *TR*, TightRope; *SD*, standard deviation; *CI*, confidence interval; *ASES*, American Shoulder and Elbow Surgeons.

Two studies reported American Shoulder and Elbow Surgeons (ASES) scores at different follow-up times.^13^^,^^23^ No significant difference was found between the HP and TR groups (MD, 0.39; 95% CI, −0.90 to 1.68; *P* = .55). There was also no significant difference noted at 24 months after surgery (MD, 1.45; 95% CI, −0.28 to 3.17; *P* = .10) (Fig. 7).

### Other functional scores

One study reported no difference in Korean Shoulder Scores at different follow-up times.^13^

### Coracoclavicular distance (mm)

**Figure 8:**
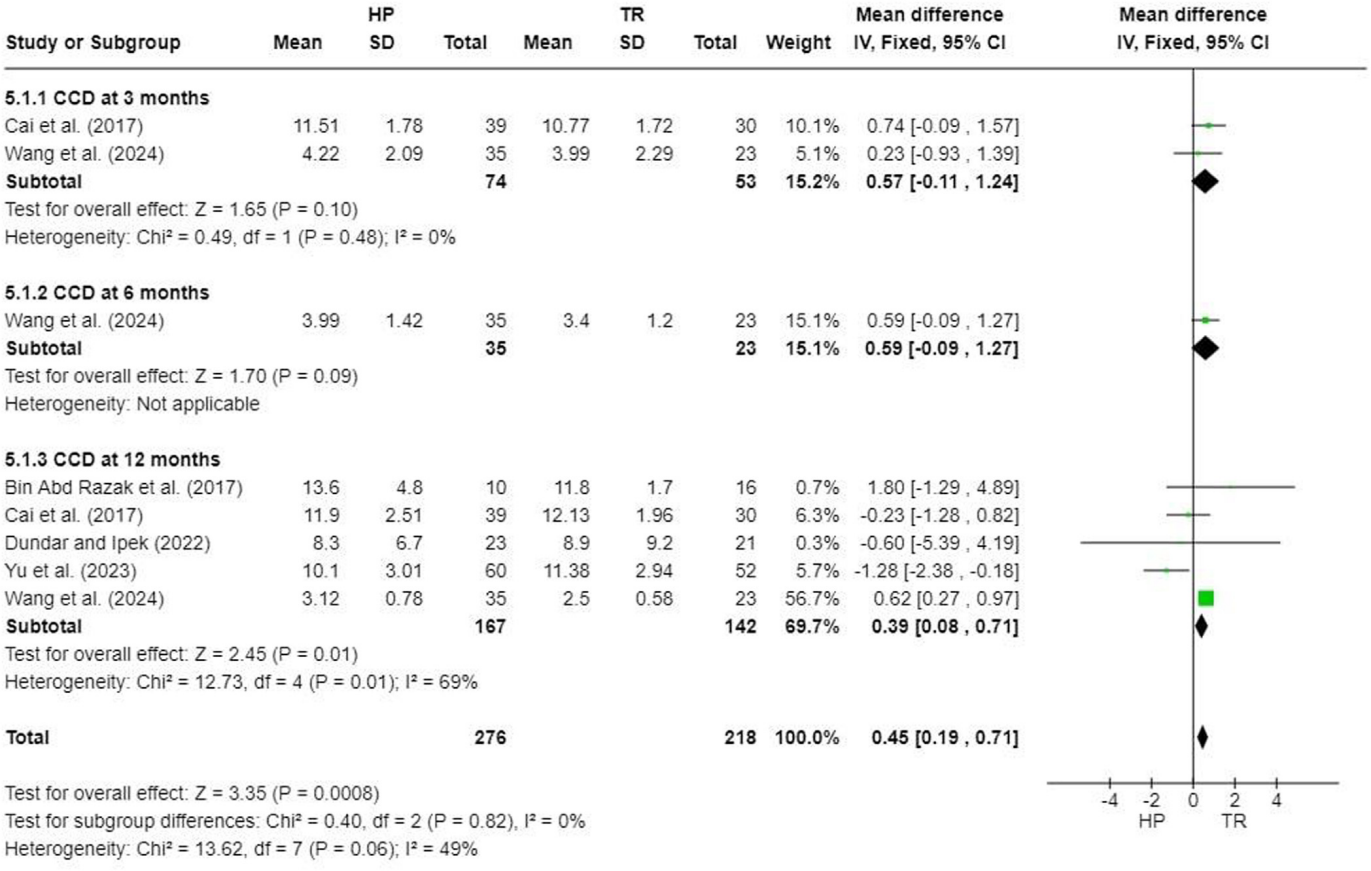
Forest plot of coracoclavicular distance (mm). *HP*, hook plate; *TR*, TightRope; *SD*, standard deviation; *CCD*, coracoclavicular distance; *CI*, confidence interval.

Seven studies reported CCD at different follow-up times,^3^^,^^5^^,^^7^^,^^8^^,^^26^^,^^27^^,^^31^ but 1 did not specify the time at which CCD was recorded,^26^ and 1 did not report standard deviations.^8^ A meta-analysis performed for 5 studies demonstrated a significant difference between the HP and TR groups (MD, 0.45; 95% CI, 0.19-0.71; *P* = .0008). There was no significant difference noted at 3 months after surgery (MD, 0.57; 95% CI, −0.11 to 1.24; *P* = .10), but a significant difference was observed at 12 months after the surgery (MD, 0.39; 95% CI, 0.08-0.71; *P* = .01) (Fig. 8).

### Complications

**Figure 9:**
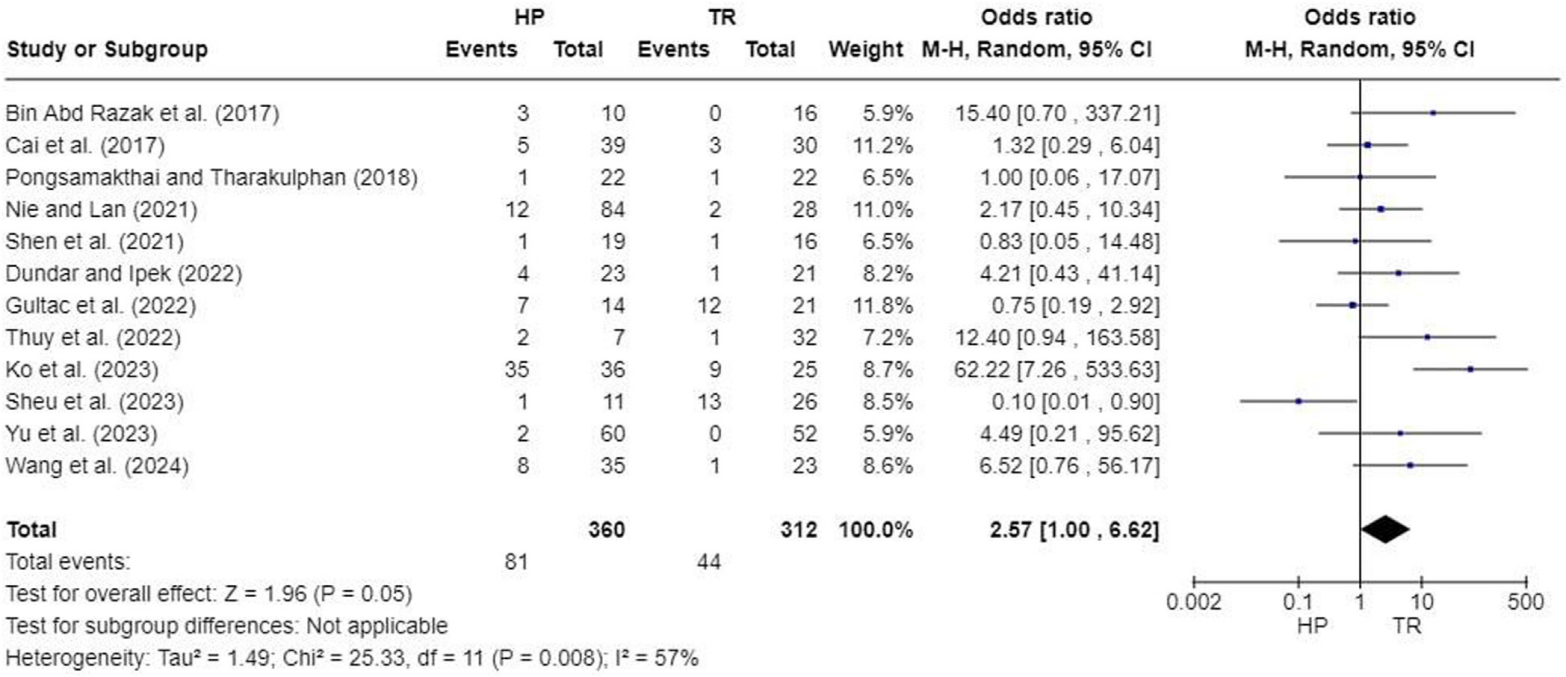
Forest plot of complication rates. *HP*, hook plate; *TR*, TightRope; *CI*, confidence interval.

**Figure 10:**
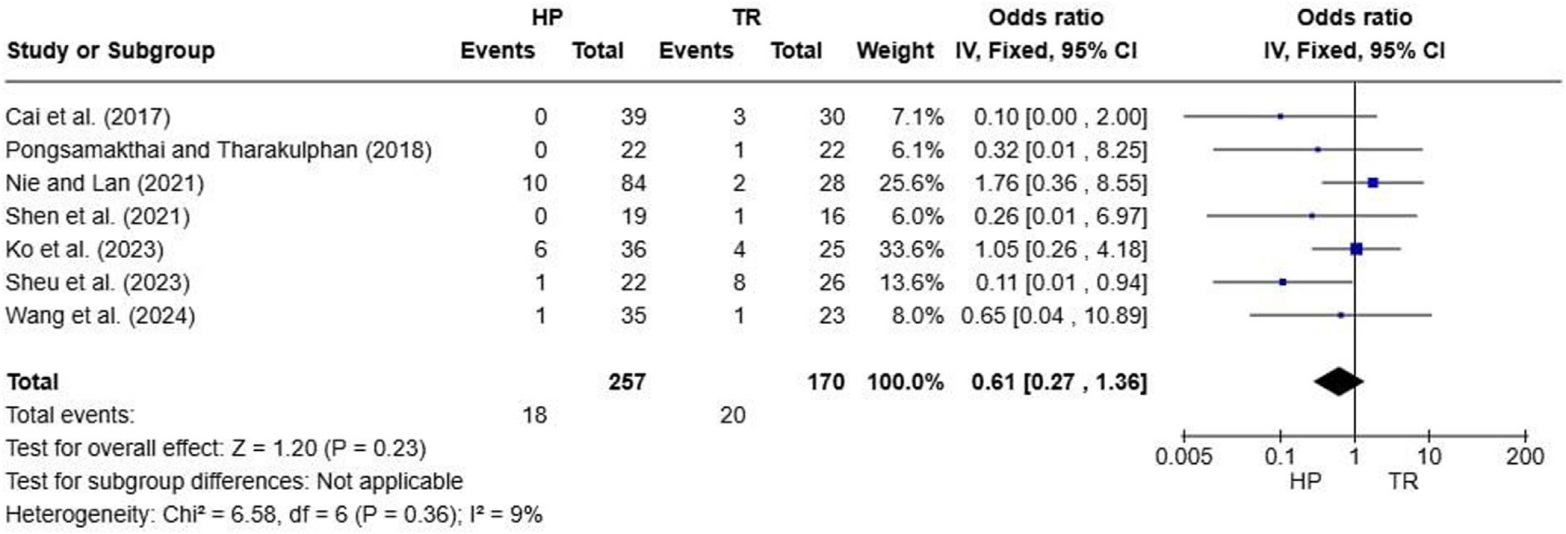
Forest plot of loss of reduction. *HP*, hook plate; *TR*, TightRope; *CI*, confidence interval.

All 12 studies reported at least 1 type of postoperative complication including acromial erosions,^3^^,^^7^^,^^8^^,^^13^^,^^20^^,^^22^^,^^27^ infections,^5^^,^^16^^,^^27^^,^^31^ arthritis,^13^^,^^27^ and loss of reduction.^5^^,^^13^^,^^16^^,^^20^^,^^22^^,^^23^^,^^27^ A meta-analysis of total complication rates demonstrated no significant difference in complication rates (OR, 2.57; 95% CI, 1.00-6.62; *P* = .05) (Fig. 9). Acromial erosions, arthritis, and infections, superficial or deep, were only reported in HP fixation groups so specific meta-analyses for these complications were not carried out. Regarding loss of reduction, a meta-analysis performed for 7 studies demonstrated no significant difference in complication rates (OR, 0.61; 95% CI, 0.27-1.36; *P* = .23) (Fig. 10).

### Quality assessment

Of the 12 included studies, 2 (16.7%) were RCTs which were evaluated using the Cochrane risk of bias tool (Table II). Both studies carried out randomization using sealed envelopes and neither of them reported significant deviations from the intended treatment or demonstrated any bias in the selection of the reported result.^5^^,^^20^ One study appropriately utilizes the intention-to-treat principle for patients lost to follow-up, to address missing data but does not specify whether assessors were blinded or not.^20^ The other study did not state how missing outcome data were managed and minimized assessor bias by utilizing 2 examiners who were not involved in the surgical treatment of patients.^5^

**Table II:** Cochrane Risk of Bias 2 score for randomized control studies.

Author (yr)	Bias arising from randomization	Bias due to deviations from intended intervention	Bias due to missing outcome data	Bias in the measurement of the outcome	Bias in the selection of the reported result.	Overall bias
Cai et al (2017)^5^	Low	Low	Unclear	Low	Unclear	Unclear
Pongsamakthai and Tharakulphan (2018)^20^	Low	Low	Low	Unclear	Low	Unclear

*HP*, hook plate; *NA*, not applicable; *SD*, standard deviation; *TR*, tightrope.

The remaining 10 (83.3%) nonrandomized studies were assessed using the MINORS tool (Table I). Nonrandomized studies can be further divided into noncomparative and comparative studies. The mean MINORS score was 17.8 ± 2.7, indicating that the comparative possessed a good quality of evidence. All 10 of the included studies reported a clearly stated aim, appropriate endpoints, and appropriate follow-up periods. Comparison groups were also appropriate, contemporaneous, and possessed similar baseline characteristics. However, only 2 studies reported prospective data collection,^3^^,^^16^ 4 studies reported a <5% loss to follow-up,^3^^,^^16^^,^^27^^,^^31^ and 4 studies performed a power analysis.^3^^,^^13^^,^^23^^,^^27^

## Discussion

Overall, our meta-analysis used several outcome measures to compare HP and TR fixation in ACJ dislocations. CMS scores (MD, −3.56; 95% CI, −5.37 to −1.75; *P* = .0001) and intraoperative blood losses (MD, 41.27; 95% CI, 30.67-51.87; *P* < .00001) indicated advantages for the HP group over the TR group. VAS scores (MD, 0.55; 95% CI, 0.34-0.76; *P* < .0001), and postoperative CCD (MD, 0.45; 95% CI, 0.19-0.71; *P* = .0008) indicated advantages for the TR group over the HP group. Operative time (MD, 1.75; 95% CI, −16.55 to 20.05; *P* = .85), UCLA scores (MD, 0.34; 95% CI, −0.81 to 1.48; *P* = .56), ASES scores (MD, 0.39; 95% CI, −0.90 to 1.68; *P* = .55), and complications (OR, 2.57; 95% CI, 1.00-6.62; *P* = .05) were similar between HP and TR groups.

A previously published meta-analysis of 4 studies by Pan et al. concluded that the TR had significantly lower VAS pain scores than the HP, and there were no significant differences in CMS, UCLA, CCD and complication rates.^19^ This meta-analysis however included 1 study where the 2 groups could not be statistically compared,^1^ and another which compared the double TR technique with the HP,^12^ 2 studies which we chose to exclude in our meta-analysis. Furthermore, our meta-analysis includes 10 more studies that were published between 2020 and 2024.^7^^,^^8^^,^^13^^,^^16^^,^^20^^,^^22^^,^^23^^,^^26^^,^^27^^,^^31^ Our meta-analysis also concluded that VAS pain scores favored the TR. Furthermore, rather than comparing the 2 techniques, a biomechanical study concluded that combining them achieved greater fracture stability and could increase fracture healing rates.^21^

Regarding other techniques, a meta-analysis by Gupta et al. comparing the HP with the double EndoButton concluded that the double EndoButton had better functional and clinical outcomes but had higher implant failure rates while HPs had a higher rate of complications like subacromial erosion, ACJ arthrodesis, and infection.^9^ A network meta-analysis comparing HPs, TR, EndoButton, tendon grafts and suture anchors concluded that the HP showed less CMS improvement than the TR with no significant differences in CCD, and that HPs had the highest incidence of complications.^30^ Liu et al. reported better functional recovery at early follow-ups with a modified minimally invasive TR loop plate technique than the traditional open reduction with HP fixation in the management of Rockwood Type III ACJ dislocations.^15^

Surveys have also found that the choice of technique may depend on the surgeon's number of years of experience and specialty training, 1 survey carried out in Germany reporting that nonspecialists prefer the HP while specialists prefer the arthroscopic TR technique,^2^ and another survey done in the United Kingdom reporting that younger surgeons with <10 years of practice employ a greater variety of techniques, even those which are new and lacking evidence of superior results, while older and more experienced surgeons are more likely to rely on reproducible and well-established techniques.^6^

The main strength of this meta-analysis is its methodology. Four databases were screened and included references were hand searched to identify relevant studies. Furthermore, screening, data extraction, and quality assessment were completed by 2 independent reviewers. The sample size of 683 included patients allowed the comparison of clinical outcomes and complications across diverse populations from 7 different countries. However, this meta-analysis is not without limitations. Although our literature search utilized 4 databases, there may have been potentially relevant studies indexed on other databases that may have been missed out from our search strategy. Another major limitation is that our review only included 2 RCTs. As such, future RCTs on this subject would aid in increasing the validity of our findings. Postoperative outcomes were also reported using 5 different scoring tools, with either modality scoring better depending on the tool used, making it difficult to compare results across all studies. Included studies also reported on a wide range of complications but incident rates were not always specified, making it difficult for a meta-analysis of each specific complication to be carried out.

## Conclusion

TR fixation in ACJ dislocations had similar operative times, complication rates, UCLA scores, and ASES scores to HP fixation. The HP had less intraoperative blood loss and better CMS scores. Conversely, TR fixation had better VAS scores and smaller postoperative CCD. Future RCTs on this subject would aid in increasing the validity of our findings.

## Disclaimers

Funding: No funding was disclosed by the authors.

Conflicts of interest: The authors, their immediate families, and any research foundations with which they are affiliated have not received any financial payments or other benefits from any commercial entity related to the subject of this article.
